# Caveolin-1 promotes glioma progression and maintains its mitochondrial inhibition resistance

**DOI:** 10.1007/s12672-023-00765-5

**Published:** 2023-08-29

**Authors:** Yu’e Liu, Yi Chen, Fei Wang, Jianghua Lin, Xiao Tan, Chao Chen, Lei-lei Wu, Xiaoling Zhang, Yi Wang, Yufeng Shi, Xiaoli Yan, Kaijun Zhao

**Affiliations:** 1 Department of Neurosurgery, Shanghai East Hospital, School of Medicine, Tongji University, Shanghai, 200120 China; 2 The China-US (Henan) Hormel Cancer Institute, Zhengzhou, 450000 China; 3 Shanghai Pudong Hospital, Pudong Medical Center, Fudan University, Shanghai, 201399 China; 4 Tongji University Cancer Center, Shanghai Tenth People’s Hospital of Tongji University, School of Medicine, Tongji University, Shanghai, 200092 China; 5 Department of Neurosurgery, Changhai Hospital, No. 168 Changhai Road, Shanghai, 200433 China; 6 Department of Thoracic Surgery, Shanghai Pulmonary Hospital, School of Medicine, Tongji University, Shanghai, 200433 China; 7 National Joint Engineering Laboratory for Human Disease Animal Models, First Affiliated Hospital of Jilin University, Changchun, China; 8 Key Laboratory of Organ Regeneration and Transplantation, First Hospital of Jilin University, Changchun, China; 9 Department of Critical Care Medicine, Sichuan Academy of Medical Science and Sichuan Provincial People’s Hospital, University of Electronic Science and Technology of China, Chengdu, China; 10 Clinical Center for Brain and Spinal Cord Research, Tongji University, Shanghai, 200092 China; 11 Laboratory of Immunology and Pathogen Biology, School of Medicine, Tongji University, Shanghai, 200092 China

**Keywords:** CAV1, DNA methylation, Drug resistance, Glioma, Immunotherapy

## Abstract

**Background:**

Glioma is a lethal brain cancer and lacking effective therapies. Challenges include no effective therapeutic target, intra- and intertumoral heterogeneity, inadequate effective drugs, and an immunosuppressive microenvironment, etc. Deciphering the pathogenesis of gliomas and finding out the working mechanisms are urgent and necessary for glioma treatment. Identification of prognostic biomarkers and targeting the biomarker genes will be a promising therapy.

**Methods:**

From our RNA-sequencing data of the oxidative phosphorylation (OXPHOS)-inhibition sensitive and OXPHOS-resistant cell lines, we found that the scaffolding protein caveolin 1 (CAV1) is highly expressed in the resistant group but not in the sensitive group. By comprehensive analysis of our RNA sequencing data, Whole Genome Bisulfite Sequencing (WGBS) data and public databases, we found that CAV1 is highly expressed in gliomas and its expression is positively related with pathological processes, higher CAV1 predicts shorter overall survival.

**Results:**

Further analysis indicated that (1) the differentiated genes in CAV1-high groups are enriched in immune infiltration and immune response; (2) CAV1 is positively correlated with tumor metastasis markers; (3) the methylation level of CAV1 promoters in glioma group is lower in higher stage than that in lower stage; (4) CAV1 is positively correlated with glioma stemness; (5) higher expression of CAV1 renders the glioma cells’ resistant to oxidative phosphorylation inhibitors.

**Conclusion:**

Therefore, we identified a key gene CAV1 and deciphered its function in glioma progression and prognosis, proposing that CAV1 may be a therapeutic target for gliomas.

**Graphical Abstract:**

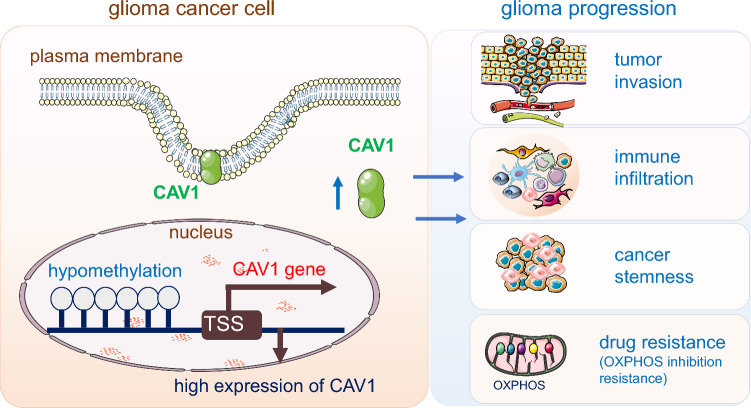

**Supplementary Information:**

The online version contains supplementary material available at 10.1007/s12672-023-00765-5.

## Introduction

Gliomas are the most commonly malignant and primary brain tumors with significant mortality and morbidity [1], counting for 81% of malignant intracranial tumors. The World Health Organization (WHO) divided gliomas into four grades: grade I and grade II are the low-grade gliomas (LGG) and have fewer malignant characteristics than the higher-grade gliomas (WHO grade III and grade IV), the grade IV gliomas named glioblastoma multiforme (GBM) is the most lethal one, and has a 5-year relative survival of ∼5% [2–4]. Standard treatment of gliomas includes surgical resection followed by radiation therapy and chemotherapy [5]. Even the advanced technologies and immunotherapy are developing fast, to date, the medieval survival time of gliomas hasn’t improved significantly. Challenges include no effective therapeutic target, intra- and intertumoral heterogeneity, inadequate effective drugs, and an immunosuppressive microenvironment, etc. [6]. Therefore, deciphering the pathogenesis of gliomas and finding out the working mechanisms are urgent and necessary for glioma treatment.

Cancer cells acquire energy resources via glycolysis and/or oxidative phosphorylation (OXPHOS), energy metabolism reprogramming is an emerging hallmark of cancer and targeting cancer cell energy metabolism has become a novel therapy [7–9]. Glioma tumor growth consumes energy and resources, in the process of neoplastic transformation, tumor initiation and progression, metabolism is significantly altered. The glycolysis was highly activated and lactate production was strongly increased regardless of oxygen availability in tumor microenvironment (TME), which is known as the Warburg effect [10]. This aerobic glycolysis potentiates glioma tumor immune evasion by hexokinase-2 mediated phosphorylation of IκBα [11]. The high reliance on glycolysis of glioma bulk cells restricted the application of OXPHOS-inhibition drugs. According to our analysis of the OXPHOS-inhibition resistant and OXPHOS-inhibition sensitive cancer cells [12], we found the scaffolding protein encoded by Caveolin-1(CAV1) is highly expressed in the OXPHOS-inhibition resistant cells lines, thus we postulate that CAV1 may function in glioma and contributes to its resistance to OXPHOS inhibition.

CAV1 is one of the main components of the caveolae plasma membranes in most cell types. It is highly involved in the modulation of cancer cell glycolysis [13], for example, it protects colon cancer cells from apoptosis by increasing the cancer cells’ glycolysis [14]. CAV1 deficiency reduced glucose uptake, lactate production and intracellular ATP output [15]. Conversely, CAV1 activation enhances glucose uptake, lactate production and cell proliferation and these effects are positively correlating with hexokinase 2 (HK2) and Recombinant Glucose Transporter 3 (GLUT3, also known as SLC2A3) expression [16]. CAV1 serves as the docking site for glycolytic enzymes, phosphofructose kinase (PFK, a rate-limiting glycolytic enzyme) co-localized with CAV1 through binding to its scaffolding domain [17]. CAV1 also plays a critical role in immune response as it works with the T cell antigen receptor (TCR) and the B cell antigen receptor (BCR) in their reorganization upon activation. After allogeneic hematopoietic cell transplantation, CAV1 expression increased in human and murine T cells, it modulates TCR signal strength and regulatory T-cell (Treg) differentiation [18]. CAV1-deficient mice exhibit defective innate immunity and inflammatory immune response under the bacterial infection [19]. However, the function of CAV1 in gliomas hasn’t been explored.

In this research project, we comprehensively analyzed the relationship between the expression of CAV1 and glioma patients’ survival, the immune cell infiltration features, the CAV1 methylation status and its functions in glioma progression and cancer cells’ OXPHOS inhibition based on different databases, either from public resources or our own RNA-sequencing and WGBS sequencing.

## Materials and methods

### Data collection

1. The RNA-seq and Whole Genome Bisulfite Sequencing (WGBS) raw sequence data reported in this paper has been deposited into the Genome Sequence Archive (GSA) for humans under accession: HRA001452. They can be downloaded by accessing https://ngdc.cncb.ac.cn/search/?dbId=hra&q=HRA001452&page=1.
2. cRNA-seq data of glioma tumor tissues and normal tissues were obtained from The Cancer Genome Atlas (TCGA) https://portal.gdc.cancer.gov/, and verified from the Gene Expression Omnibus (GEO, https://www.ncbi.nlm.nih.gov/geo/) and the Chinese Glioma Genome Atlas (CGGA) http://www.cgga.org.cn/.

### mRNA and protein expression of CAV1

The UALCAN database (ualcan.path.uab.edu/index.html) was checked to analyze relationships of CAV1 mRNA expression. The protein expression of CAV1 was obtained from the human protein atlas (HPA) (http://www.proteinatlas.org/) database.

### Survival and statistical analysis

To investigate whether CAV1 expression level affects the clinical outcomes of Glioma patients, we divided the cancer samples into two groups according to the median mRNA expression value of CAV1(CAV1-high and -low groups) and then we constructed a prognostic classifier by checking Kaplan–Meier (KM) survival curves to compare the survival disparities (https://kmplot.com/analysis/).

### Univariate and multivariate logistic regression analysis

To further determine the effect of CAV1 expression in glioma patients, the univariate Cox regression analysis was used to calculate the association between the expression level of CAV1 and the patients’ overall survival (OS) by R package “survival”. Afterward, we used a multivariate analysis to evaluate whether the CAV1 is an independent prognostic marker for glioma patient survival.

### CAV1 protein interaction analysis

We explored the interaction protein of CAV1 via the the STRING database (https://cn.string-db.org/).

### Identification and enrichment analysis of DEGs in CAV1-high and-low groups

Distinct CAV1 subtype-related differentially expressed genes (DEGs) were identified using the “limma” package in R (adj. p < 0.05 and |log_2_FC|> 2) [20]. The functional and enrichment pathways (GO and KEGG) of DEGs were further explored using the “cluster profiler” package in R [21].

### Function and pathway analysis by Gene Set Enrichment Analysis (GESA)

DEGs between CAV1-high and low groups were identified by using the DESeq 2 R package. GSEA was performed using the ggplot2 R package to explore the significant functions and pathways between the two groups. CAV1 expression was used as a phenotype label. An adjusted p-value (adj.p) < 0.05, normalized enrichment score(|NES|) > 1.5, and false discovery rate (FDR) < 0.05 were considered significant difference.

### The combination analysis of GO /KEGG and LogFC

We get the relations between the expression of CAV1 and the biological process (BP), cellular component (CC) and molecular function (MF) via GO analysis, then we conduct KEGG pathway analysis. On the basis analysis of GO and KEGG analysis, with the data of logFC, we calculated the zscore of each item.

$$\text{zscore}=\frac {\text{Up}-\text{Down}}{\sqrt{\text{Counts}}}$$

### Immune cells infiltration analysis

We checked the relationship between the CAV1 and the respective abundance of infiltrating immune cells (macrophages, CD4^+^ T cells, CD8^+^ T cells, B cells, neutrophils, and dendritic cells) in glioma patients by The Tumor Immune Estimation Resource (TIMER) algorithm database (https://cistrome.shinyapps.io/timer/).

### The correlation between the expression of CAV1 and the infiltration of immune cells

The tumor immune infiltration analysis with more immune cells (total 22) were analyzed by ssGSEA by GSVA R package. Original data is from TCGA (https://portal.gdc.cancer.gov/).

### Estimation of stromal and immune cells in malignant tumors using expression data (ESTIMATE)

The Estimation of Stromal and Immune Cells in Malignant Tumors using Expression Data (ESTIMATE) is a package which uses gene expression data to predict the content of interstitial cells and immune cells in malignant tumor tissues [22]. Based on the enrichment analysis of a single sample gene set (ssGSEA), the algorithm generates three scores: stromal score (recording the presence of stroma in tumor tissue), immune score (representing the infiltration of immune cells in tumor tissue), estimated score (inferring tumor purity). The Stromal score, Immune Score and Estimate Score of CAV1 can be obtained at http://www.sangerbox.com/.

### The correlation between CAV1 expression and the immune checkpoint

The pan-cancer data were downloaded from UCSC (https://xenabrowser.net/) including the TCGA TARGET GTEx, then we checked the expression of Pearson correlation between the expression of ENSG00000105974 (CAV1) and 60 immune checkpoints including 24 inhibitory and 36 stimulatory immune checkpoints [23].

### The DNA methylation of CAV1

The DNA methylation of CAV1 in glioblastoma for 659 samples from TCGA was checked from website: https://mexpress.be/. The cluster analysis of CAV1 DNA methylation for low grade glioma and glioblastoma multiforme was obtained from below website: http://bio-bigdata.hrbmu.edu.cn/diseasemeth/.

### The correlation between the expression of CAV1 and its methylation

Epigenome-Wide Association Study (EWAS) has become a standard strategy to discover DNA methylation variation of different phenotypes [24]. The methylation level of CAV1 gene in gliomas and the methylation changing with the expression of CAV1 were explored at EWAS by accessing https://ngdc.cncb.ac.cn/ewas/datahub/. All the relations between methylation of CAV1 and patients survival were checked from http://www.cgga.org.cn/.

### The correlation between the methylation of CAV1 and patients’ survival

MethSurv (https://biit.cs.ut.ee/methsurv/) is a network tool for survival analysis based on the CpG methylation model. It uses 7358 methylation data from 25 different human cancers from the TCGA database and uses Cox proportional risk model to develop an interactive network tool for survival analysis. We checked the correlation between the DNA methylation of CAV1 and patients’ survival from this website.

### Relationship between CAV1 gene expression and cancer stemness

We checked the Pearson correlation between the expression of CAV1 and cancer stemness in the pan-cancer atlas by accessing http://sangerbox.com. The stemness was calculated based on the RNA-based stemness scores derived by the stemness group, the DNA methylation based stemness scores derived by the stemness group and other stemness probes (219 probes).

### The expression of CAV1 in scRNA-seq

Tumor Immune Single-cell Hub 2 (TISCH2) is a scRNA-seq database, which aims to characterize tumor microenvironments at single-cell resolution. TISCH2 (http://tisch.comp-genomics.org) has collected 187 sets of high-quality tumor single cell transcriptome data and corresponding patient information from GEO and ArrayExpress [25]. The data covers 50 cancer types, including 6 million cells from more than 1500 patients. Among them, 40 sets of TISCH2 data are single cell transcriptome data under different treatment conditions, including immunotherapy, chemotherapy, targeted therapy and combination therapy.

### The oligomycin A sensitivity data for glioma cells

Data of glioma cells’ viability were obtained from the cancer Dependency Map (DepMap) portal (https://depmap.org/portal/). The data of DepMap mainly comes from Cancer Cell Line Encyclopedia (CCLE) (https://sites.broadinstitute.org/ccle), Achilles (whole genome Crispr Screen) and profiling relative inhibition simultaneously in mixtures (PRISM). The PRISM Repurposing dataset uses pooled-cell line chemical-perturbation viability to screen small molecules [26]. The viability score above 0 indicates this cell line is resistant to the drug. Cancer cell lines are from CCLE. We checked the glioma cancer cells’ viability after treatment with Oligomycin A (an OXPHOS inhibitor, targeting complex V of mitochondria).

### The CRISPR result of CAV1-knockout in different glioma cell lines

The cell viability of different glioma cell lines by knocking out CAV1 were downloaded from the Achilles DepMap datasets (https://depmap.org/portal/). CAV1 knocking down (RNAi) or knocking out (CRISPR) result were checked in DEMETR2 and CERES, respectively. The scores evaluate the effect of knocking down or knocking out CAV1 while normalizing expression against the distribution of pan-essential and nonessential genes. Positive scores (> 0) indicate that the cell line would grow faster while the negative scores indicate that the cell line would grow slower after experiment manipulation.

### Cell culture

G-401, NCI-H82, SW48, MDA-MB-453 were bought from ATCC, and WSU-DLCL2 was bought from Deutsche Sammlung von Mikroorganismen und Zellkulturen (DSMZ), SF126 cell was bought from JCRB Cell Bank. 786-O, NCI-H82, WSU-DLCL2, G-401 were cultured in 1640 medium adding 10% FBS, 1% Pen/Strep. SF-126, SW48, and HEK293T were cultured in DMEM medium adding 10% FBS, 1% Pen/Strep. CFPAC-1 was cultured in IMDM medium adding 10% FBS, 1% Pen/Strep. MDA-MB-453 was cultured in L-15 medium adding 10% FBS, 1% Pen/Strep. MDA-MB-453 was maintained in 100% air without CO_2_ at 37 °C and other cells were in 5% CO_2_ at 37 °C.

### Cell viability assay

500–1000 cells per well were seeded in 96-well plate and incubated with or without Gboxin for 72 h. Cell viability was tested by the CellTilter Glo Luminescent Cell Viability Assay (Promega, G7572).

### Preparation of RNA-Seq library

Total RNA was extracted from 1 × 10^6^ cells for each sample. RNA integrity number (RIN) higher than 7.0 was used as the criteria for library preparation. TruSeq Stranded Total RNA with Ribo-Zero Gold kit (Illumina, 20020598) was used to prepare the sample; PCR was used to generate the library. Agilent Bioanalyzer 2200, 150 bp paired-end sequencing was conducted on an Illumina Novaseq 6000 sequencer for the ollowing quantification.

### Generation of WGBS library

DNA was extracted from 1 × 10^6^ cells by using Allprep DNA/RNA/Protein Mini Kit (Qiagen, 80,004). Total DNA quality was evaluated by NanoDrop 2000. High-quality DNA sample (1 µg) spiked with 26 ng unmethylated lambda DNA (Promega, D1521) was fragmented into ≈250-bp fragments by S220 Focused-ultrasonicator (Covairs). After end-repairing and dA-tailing using 5 × ER/A-Tailing Enzyme Mix (Enzymatics, Y9420L), fragmented DNAs were ligated by cytosine-methylated barcodes and treated with bisulfite with EZ DNA Methylation-Gold Kit (Zymo, D5006). The WGBS libraries were built by PCR. Following sequencing was performed in Illumina Novaseq 6000 sequencer by 150 bp pair-end protocol.

### Statistical analysis

The different expression levels of CAV1 in pan-cancers were compared by the Wilcoxon test. The survival curve was used by Kaplan–Meier plot, log rank test was applied to calculate log rank *p* value. Univariate Cox regression model was used to calculate hazard ratio (HR), 95% confidence intervals (CI) and Cox *p* values in PrognoScan. Spearman’s coefficient was used to analyze the correlation of gene expression. The receiver operating characteristic (ROC) curve of glioma patients was calculated in CAV1-high and low groups, and a ROC was generated with MedCalc in R version 4.0.2 (https://www.r-project.org/). In all the statistics, p < 0.05 was considered statistically significant.

## Results

### The mRNA expression and protein expression of CAV1

**Fig. 1:**
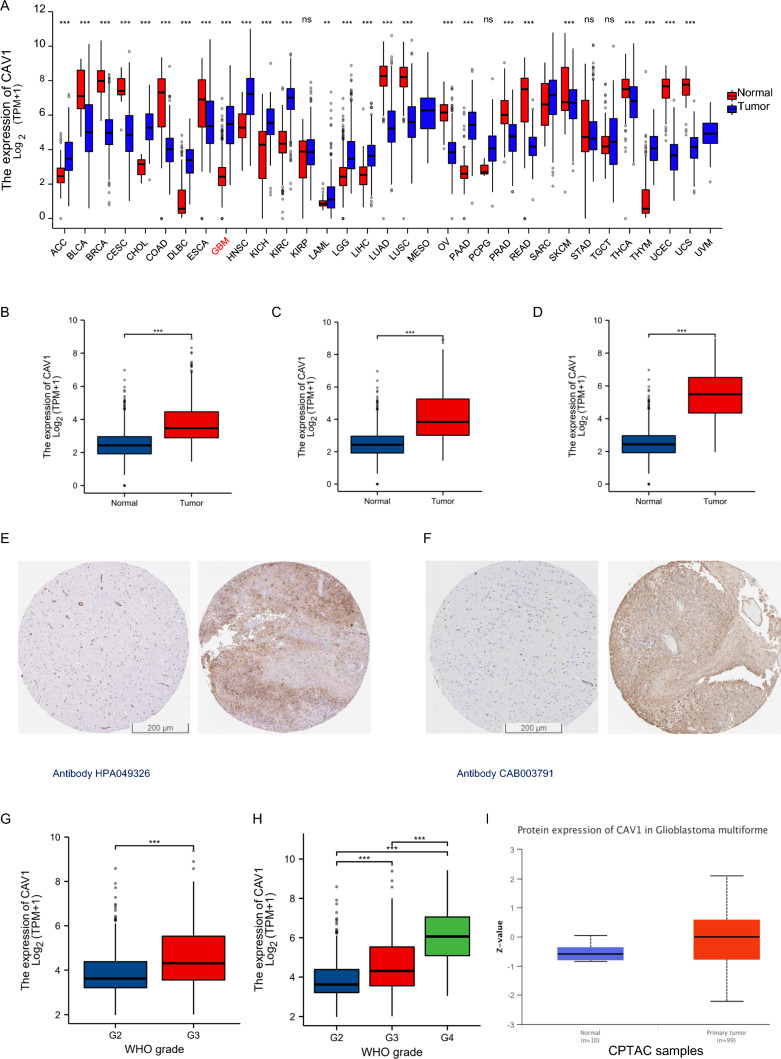

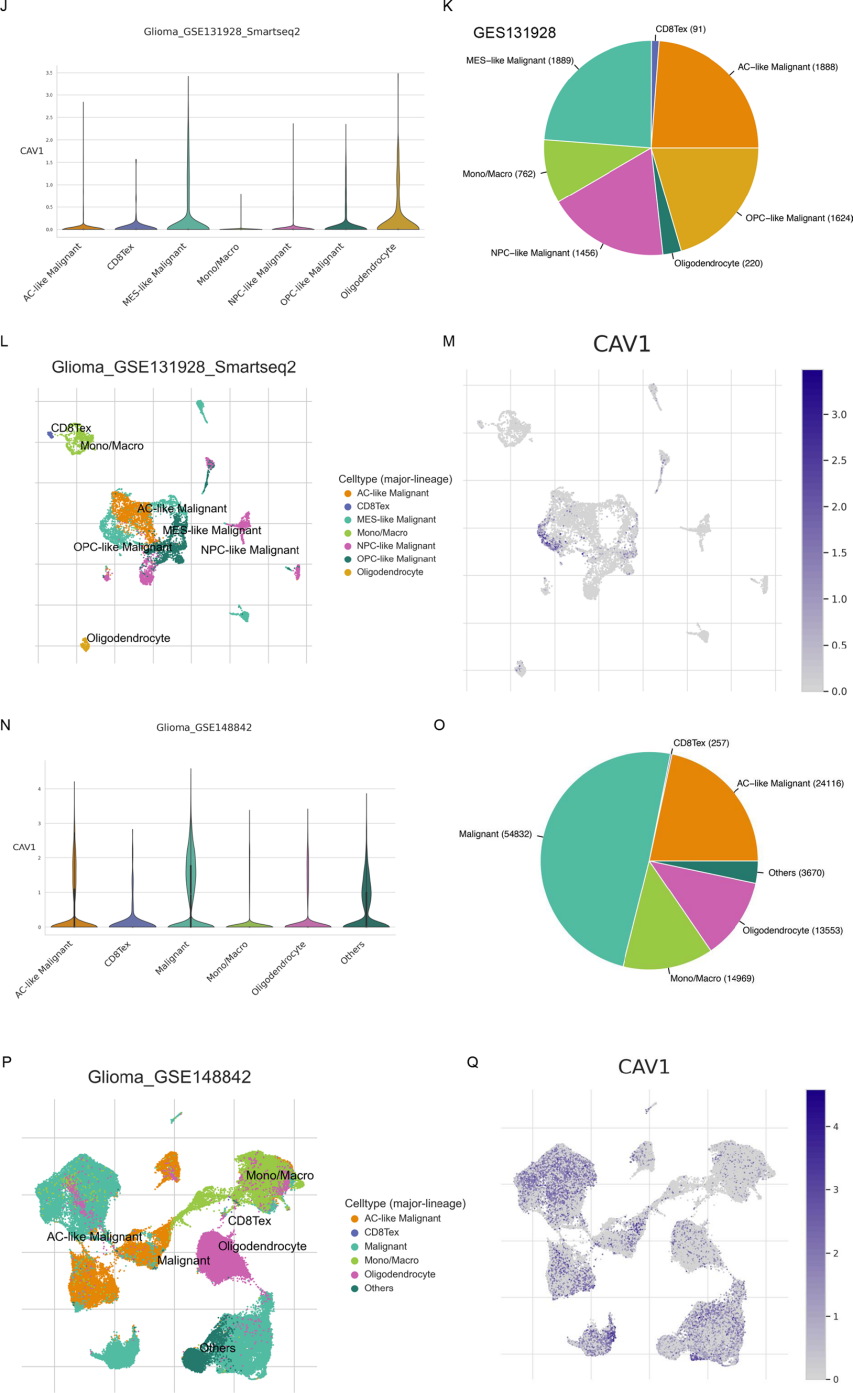
The mRNA expression and protein expression of CAV1. **A** mRNA expression of CAV1 in pan-cancers. **B** The differentiated mRNA expression of CAV1 in TCGA-LGG, total 1675 samples (1152 normal + 523 tumor samples). **C** The differentiated mRNA expression of CAV1 in TCGA-GBMLGG, total 1846 samples (1152 normal + 689 tumor samples + 5 tumor adjacent samples). **D** The differentiated mRNA expression of CAV1 in TCGA-LGG, total 1323 samples (1152 normal + 166 tumor samples + 5 tumor adjacent samples). **E** The protein expression of CAV1 in normal brain tissues (left) and glioma (right) with antibody HPA049326. **F** The protein expression of CAV1 in normal brain tissues (left) and glioma (right) with antibody CAB003791. **G**. The expression of CAV1 in different stages in LGG. **H** The expression of CAV1 in different stages of GBMLGG. **I** The protein expression of CAV1 in GBM and normal, data from UALCAN (can.path.uab.edu/). **J**–**M**. Single cell sequencing of GBM sample GSE 131928. **N**–**Q** Single cell sequencing of GBM sample GSE148842. *p value < 0.05; **p value < 0.01; ***p value < 0.001. OS: overall survival, DFS: disease-free survival

To check the CAV1 expression in different cancers, RNA sequencing data in TCGA was mined. The differential CAV1 mRNA expression between tumor and adjacent tissues were shown in Fig. 1A. CAV1 mRNA expression was significantly higher in low-grade glioma (LGG) (Fig. 1B), the combination of LGG and glioblastoma multiforme (GBMLGG) (Fig. 1C) as well as glioblastoma multiforme (GBM) (Fig. 1D), compared with adjacent tissues. CAV1 protein expression was verified by immunohistochemistry (IHC) in adjacent tissues and tumor tissues (Fig. 1E with antibody HPA049326 and Fig. 1F with antibody CAB003791). To check the relationship between CAV1 expression and the pathological progression, we checked the CAV1 expression in different stages of LGG and GBMLGG, As shown in Fig. 1G (LGG) and H (GBMLGG), the expression of CAV1 is significantly higher in the higher grade of glioma. Since GBM refers to the Grade 4 glioma, the expression of CAV1 in GBMLGG data equals the GBM data. We checked the GBM data in UALCAN database too, as shown in Fig. 1I, the protein expression of CAV1 is much higher in the GBM than that in the normal group. The expression of CAV1 is higher in glioma malignancy than other kinds of cells in the tumor microenvironment, single cell sequencing of GSE131928 (Fig. J–M,) indicates that CAV1 is highly expressed in all different malignancy cells including AC-like malignant, OPC-like malignant, MES-like malignant and NPC-like malignant cells. Another single-cell sequencing data of GSE 148842 (Fig. N–Q) indicates CAV1 is highly expressed in the malignant cells and malignant cells occupy the majority of cell lineages in all the clusters. The expression profile of CAV1 in CGGA data shows similar result as TCGA (Supplementary Fig. 1).

### The clinical correlation between CAV1 expression and glioma patients’ survival

**Fig. 2:**
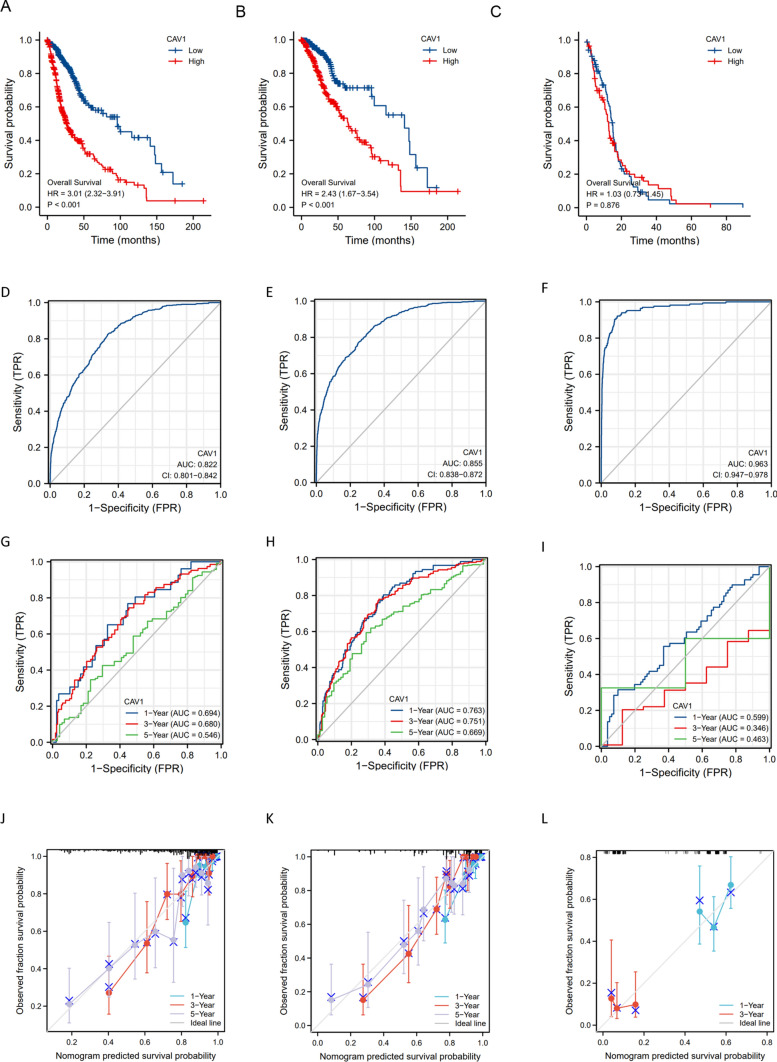

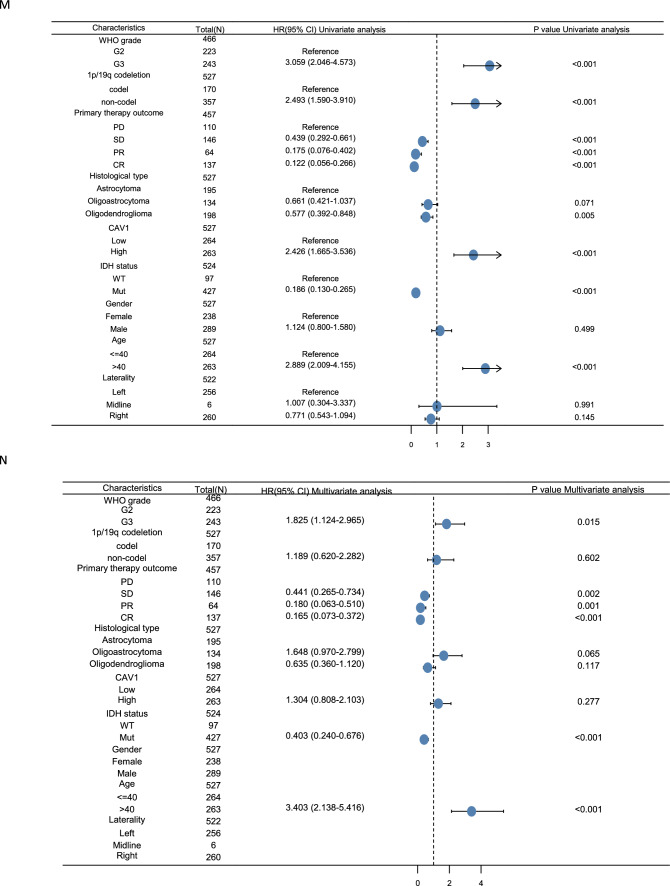
The clinical correlation of CAV1 expression in glioma. **A**–**C**. OS between and high-low expression groups of CAV1 gene in KM databases of LGG (**A**), GBMLGG (**B**) and GBM (**C**) patients. **D**–**F** ROC curve established the efficiency of CAV1 mRNA expression level on distinguishing LGG tumor (**D**), GBMLGG (**E**), and GBM (**F**) from non-tumor tissue. X-axis represents false positive rate, and Y-axis represents true positive rate. **G**–**I** The ROC curve using CAV1 as an indicator of LGG (**G**), GBMLGG (**H**) and GBM (**I**) were explored. **J**–**L** The calibration curve using CAV1 as an indicator of LGG (**J**), GBMLGG (**K**) and GBM (**L**) was checked. **M**, **N** The univariate (**M**) and multivariate regression (**N**) analysis of CAV1 and other clinicopathologic parameters with OS in LGG patients were explored. *p value < 0.05; **p value < 0.01;***p value < 0.001. OS: overall survival; DFS: disease-free survival

We explored the correlation between the high and low expression groups of CAV1 subsequently. As shown in Fig. 2A (LGG), Fig. 2B (GBMLGG), the overall survival (OS) of the CAV1-high expressed group is much shorter than that of the CAV1-low expressed group. In the GBM group in Fig. 2C, the survival difference is not as obvious as the previous that in the previous two stages.

The risk prediction model is characterized by the result of the discrimination and calibration. The receiver-operating characteristic (ROC) curve is applied for assessing the model discrimination and it is the most popular graphical method for assessing the classification accuracy of a diagnostic biomarker. It was used to analyze the effectiveness of CAV1 mRNA expression level AUC on distinguishing glioma tissues from normal issues. The AUC of CAV1 was 0.822 for LGG (Fig. 2D), 0.855 for GBMLGG (Fig. 2E), and 0.963 for GBM (Fig. 2F), suggesting that CAV1 could serve as a biomarker to distinguish glioma from non-tumor tissue. In time-to-event studies, the subject's event result is time-dependent, therefore, a new time-dependent extension of ROC curve is an important estimator. As shown in Fig. 2G–I, CAV1 as an indicator in LGG (2G), GBMLGG(2H) and GBM (2I) was explored. Calibration is a critical component for the reliability, accuracy, and precision of prediction models, we checked the calibration of CAV1 in the 1-year survival, 3-year survival and 5-year survival of LGG (Fig. 2J) and GBMLGG (Fig. 2K), for GBM (Fig. 2L), only 1-year and 3-year survival data is available, probably due to the short survival for GBM patients. The univariate (Fig. 2M) and multivariate regression (Fig. 2N) analysis of CAV1 and other clinicopathologic parameters with OS in LGG patients were explored. All the above were checked in CGGA database and similar results were shown in Supplementary Fig. 2.

### The analysis of differentiated genes in CAV1-high and -low groups in glioma patients

**Fig. 3:**
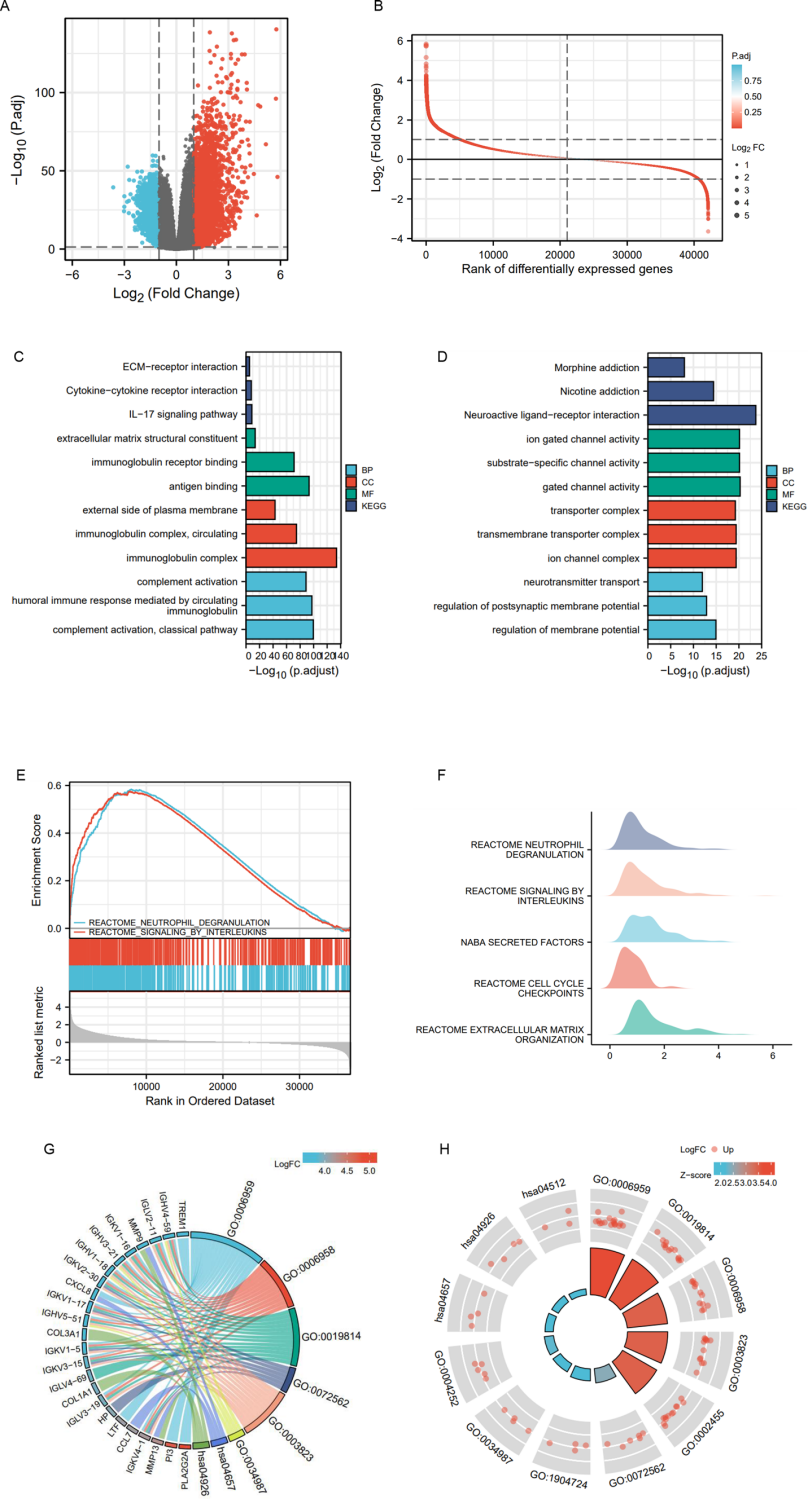
The analysis of differentiated genes in CAV1-High and -low groups in glioma patients. **A** The volcano plot shows the DEGs in CAV1-high and -low groups. **B** The Rank of differentially expressed genes. **C** The GO and KEGG analysis of high expressed genes in CAV1-high groups. **D** The GO and KEGG analysis of low expressed genes in CAV1-low groups. **E** The GSEA analysis or the DEGs. **F**–**H** The GSEA analysis in ridge plot of DEGs. **G** The combinational analysis of GO/KEGG and LogFC of CAV1-high groups

To explore the function of CAV1 in glioma, we divided the glioma patients into CAV1-high and CAV1-low groups and mined out the differentially expressed genes (DEGs) (Fig. 3A, B, Supplementary Table 1). The Gene Ontology (GO) and (Kyoto Encyclopedia of Genes and Genomes) KEGG pathway analysis (Fig. 3C) indicates that the CAV1-high groups are enriched in immunoglobulin complex, the complement activation, etc., and the CAV1-low groups are enriched in neuroactive ligand-receptor interaction (Fig. 3D). The Gene Set Enrichment Analysis (GSEA) indicates that the high expressed genes are enriched in Reactome Signaling by Interleukins and the low expressed genes are enriched in Neutrophil Degranulation (Fig. 3E, F, Supplementary Table 2). Then we conducted the combinational analysis of GO/KEGG and the LogFC. The GO enrichment and the highly expressed genes are shown in Fig. 3G and 3H (GO:0006959 humoral immune response, GO:0006958: complement activation, GO: 0019814 immunoglobin complex, GO:0003823 antigen binding).

### CAV1 expression has a positive correlation with the innate immune cell infiltration

**Fig. 4:**
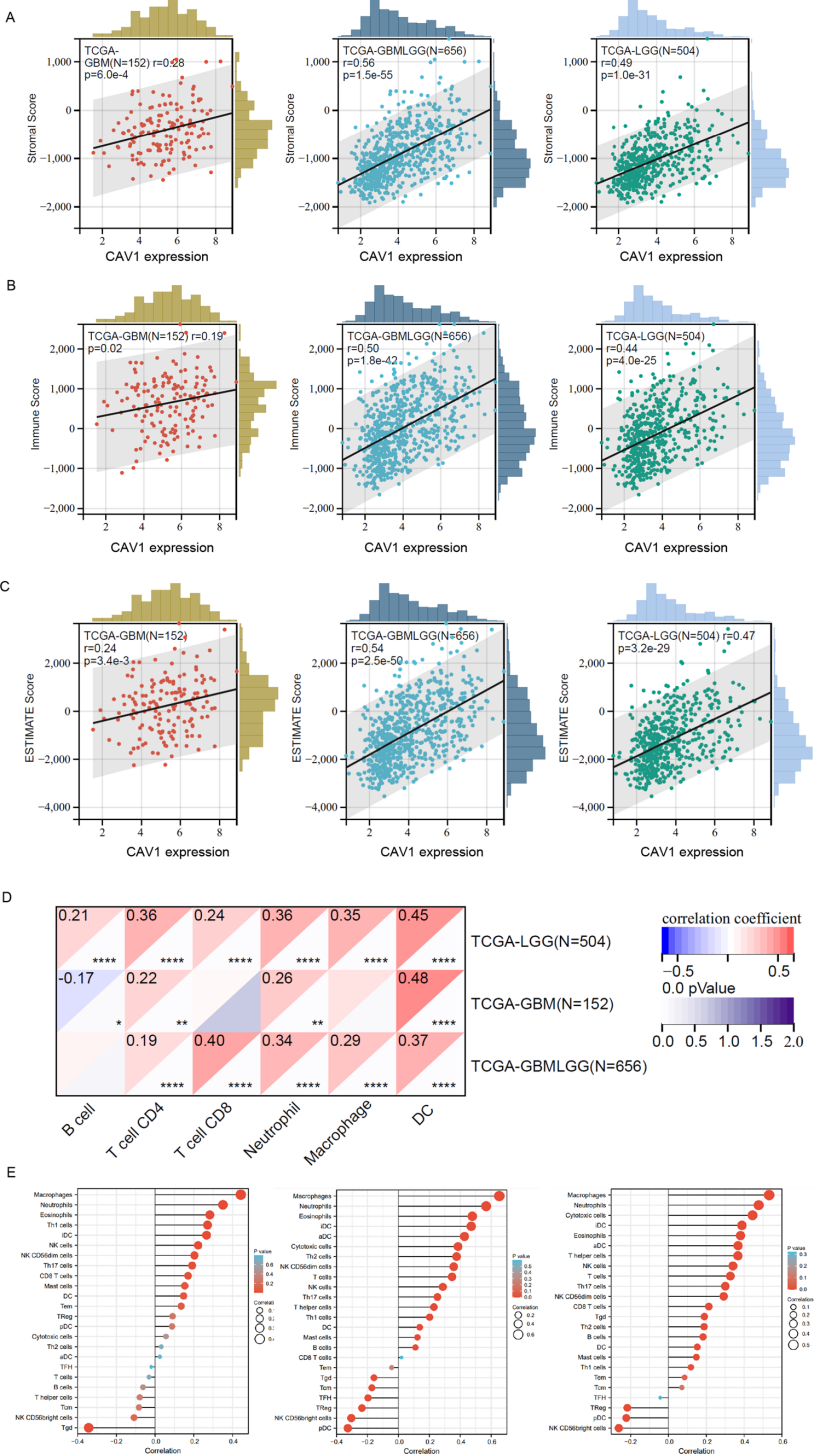

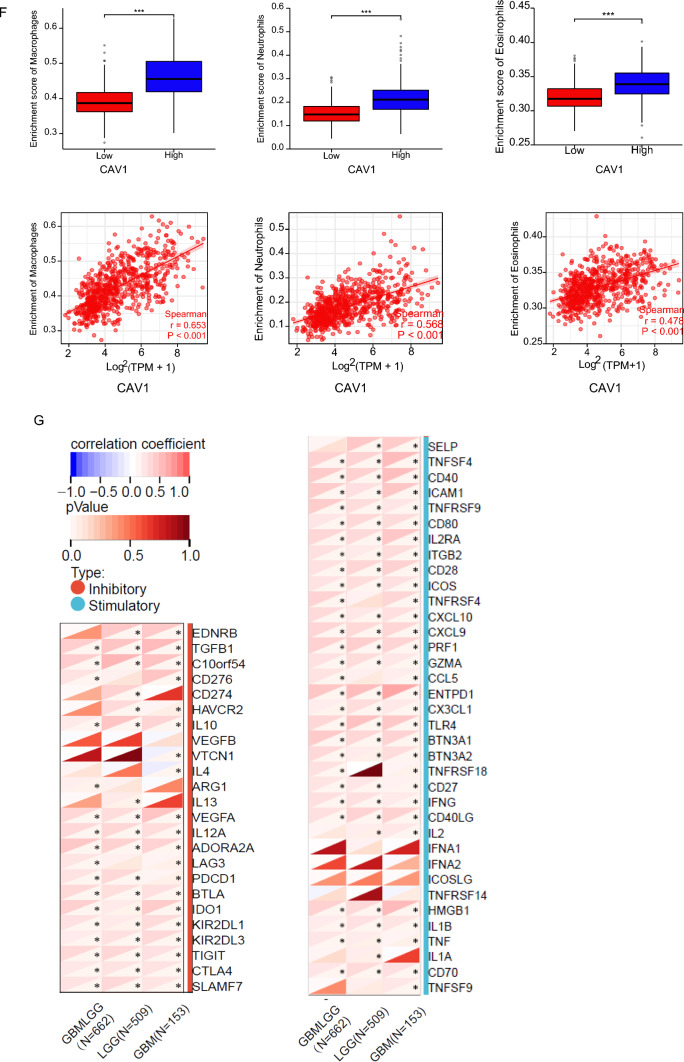
CAV1 expression has a positive correlation with the innate immune cell infiltration. **A** The correlation between the expression of CAV1 and stroma score. **B** The correlation between the expression of CAV1 and immune score. **C** The correlation between the expression of CAV1 and ESTIMATE score. **D** The correlation of the expression of CAV1 and immune cell infiltration, data from TCGA + GTEX. **E** The correlation of the expression of CAV1 and immune cells infiltration, data from GSEA via ssGESA analysis. **F**. The correlation between the expression of CAV1 and the enrichment of macrophage, neutrophil and eosinophil in GBMLGG. **G** The correlation between CAV1 expression and the immune checkpoints

Since the analysis in Fig. 3 indicated that the DEGs in CAV1-high groups are enriched in the immune response and immune infiltration, we then analyzed the expression of CAV1 and the immune infiltration in different gliomas. The stroma score and the immune score were calculated by applying Estimation of Stromal and Immune cells in Malignant Tumors using the Expression data (ESTIMATE) algorithm based on gene expression MATRIX of glioma patients in the TCGA database. Stroma scores indicate the stroma quantity in the extracellular matrix, the immune scores indicate the infiltration of immune cells in the tumor and the final ESTIMATE scores are used to deduce the tumor purity. As shown in Fig. 4A–C, the expression of CAV1 is significantly positively associated with the stroma score, immune score and ESTIMATE score (r > 0.5), and the GBMLGG type shows the highest correlation. The immune cells infiltration shows that the correlation between the expression of CAV1 and the infiltration of dendritic cells (DCs), macrophages and neutrophils are significantly based on the database from TCGA and GTEX (Fig. 4D), we then checked only TCGA via the ssGSEA analysis and found macrophages, neutrophils and eosinophils are the top infiltration immune cells and has the positive correlation with the expression of CAV1 (Fig. 4E). The expression and CAV1 and the enrichment of macrophages, neutrophils and eosinophils in GBMLGG were checked, as shown in Fig. 4F, CAV1 positively correlates with the enrichment of above immune cells. Therefore, the innate cell enrichment and infiltration in gliomas are much higher than the adaptive cells. Immune checkpoint targeting is a promising cancer immunotherapy and has been studied a lot in gliomas, the relationship between the expression of CAV1 and the immune checkpoints expression was explored. CAV1 expression is positively related with VTCN1 and TNFR18, TNFR14 in LGG patients’ samples (Fig. 4G). In a word, the immune infiltration analysis indicates that the expression of CAV1 correlates with immune infiltration positively and significantly.

### CAV1 expression positively relates to glioma cancer invasion and stemness

**Fig. 5:**
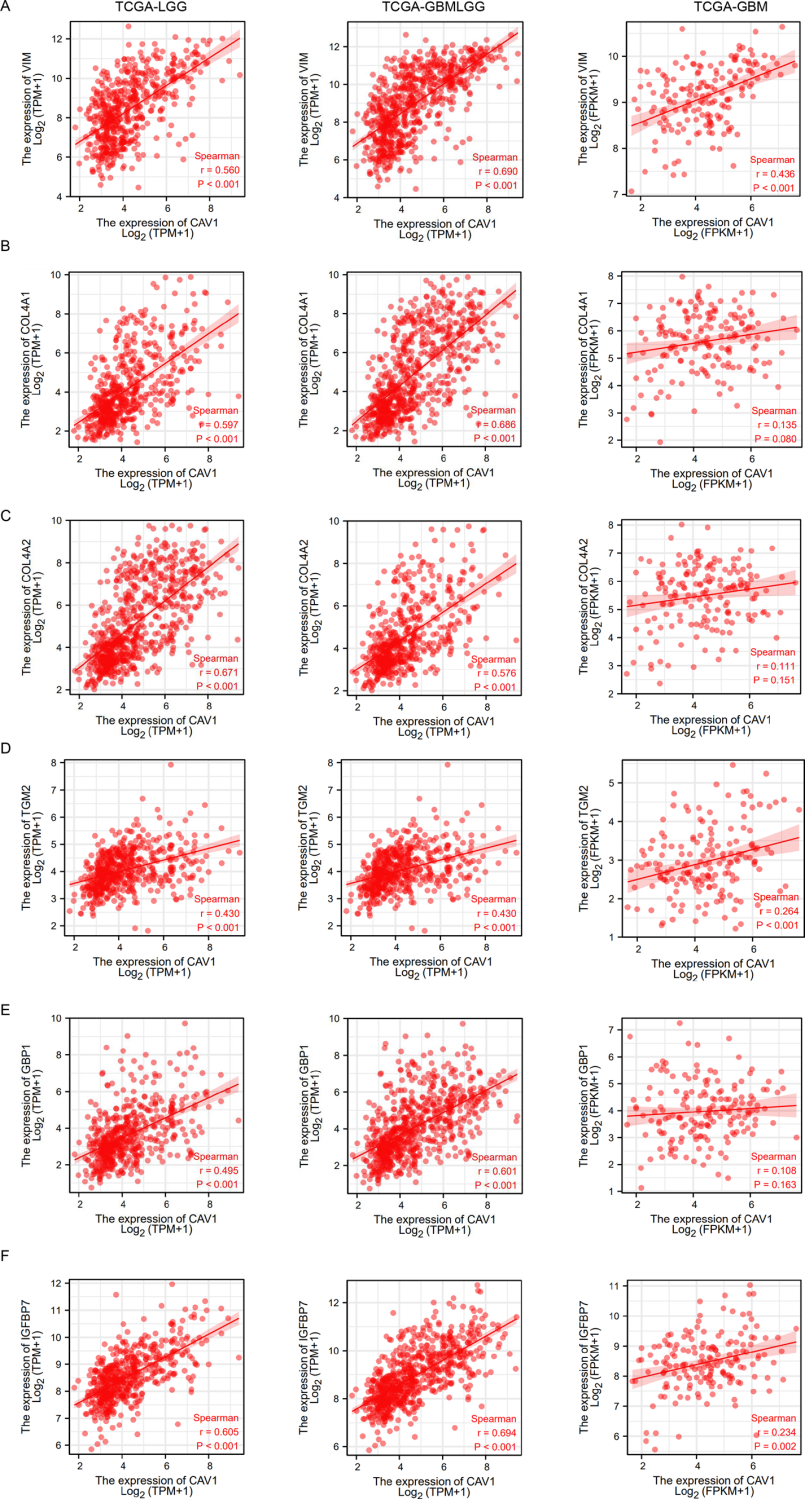

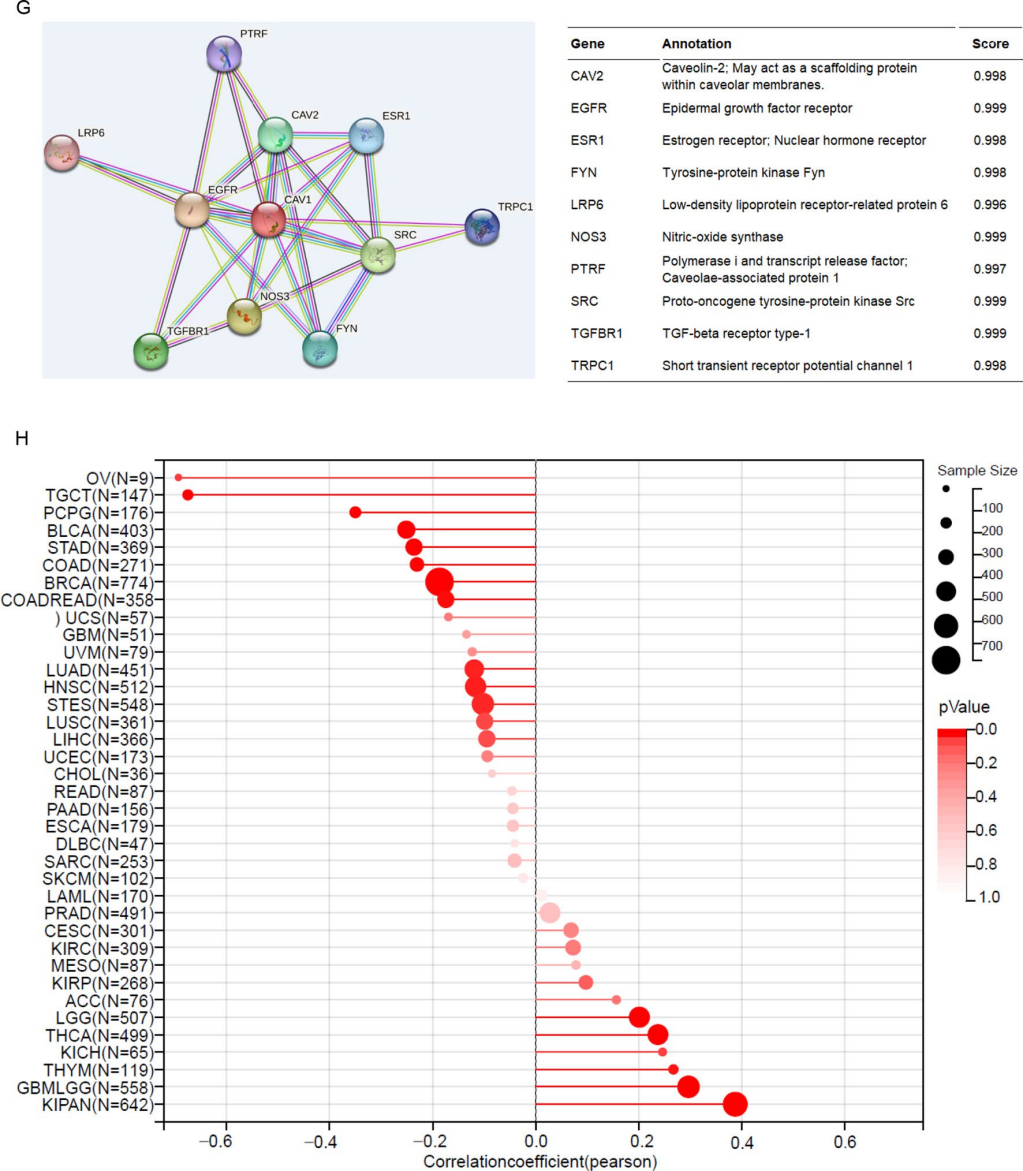
CAV1 expression is positively relates to glioma cancer cell migration. **A** The relationship between the expression of CAV1 and vimentin in LGG, GBMLGG and GBM. **B** The relationship between the expression of CAV1 and COL4A1 in LGG, GBMLGG and GBM. **C** The relationship between the expression of CAV1 and COL4A2 in LGG, GBMLGG and GBM. **D** The relationship between the expression of CAV1 and TGM2 in LGG, GBMLGG and GBM. **E** The relationship between the expression of CAV1 and GBP1 in LGG, GBMLGG and GBM. **F** The relationship between the expression of CAV1 and IGFBP7 in LGG, GBMLGG and GBM. **G** The PPI network of CAV1 was analyzed by string analysis. **H** The relationship between the expression of CAV1 and cancer stemness of pan-cancers

Since CAV1 has been demonstrated to promote pancreatic cancer invasion and metastasis [27], we would like to check its role in glioma cancer invasion and metastasis. Vimentin (VIM) is one of the human intermediate filament proteins and is required for the plasticity of mesenchymal cells under normal physiological conditions and for the migration of cancer cells that have undergone epithelial-mesenchymal transition [28]. We checked the correlation between CAV1 and VIM expression and found CAV1 expression positively significantly correlates with the expression of VIM (Fig. 5A). Besides VIM, the basement membrane also promotes cancer cell invasion and metastasis, COL4A1 and COL4A2 *ar*e typical basement membranes ubiquitously expressed on cells [29]. The expression of CAV1 positively correlates with COL4A1 (Fig. 5B) and COL4A2 (Fig. 5C). More metastasis biomarkers such as TGM2 (Fig. 5D), GBP1 (Fig. 5E) and IGFBP7 (Fig. 5F) were checked too, and all of their expressions have a positive correlation with the expression in CAV1 in all different types of gliomas. Moreover, we also checked the protein–protein interaction (PPI) network of CAV1 and found it is highly connected with Caveolin-2 and EGFR (Fig. 5G), which are important factors for tumor progression. Therefore, we propose that the high expression of CAV1 may enhance glioma cancer cell invasion and progression.

Cancer stem cells (CSCs) are self-renewing cells that facilitate tumor initiation, promote metastasis, and enhance cancer therapy resistance [30]. Glioma cancer stemness is highly related to its progression, drug resistance and recurrence. Therefore, we explored the relationship between CAV1 and glioma stemness from RNA sequencing data in TCGA and GTEX, and found that the expression of CAV1 is positively related to the glioma stemness in pan-cancer analysis (Fig. 5H).

### The methylation status of CAV1 and its relationship with patient’s survival time

**Fig. 6:**
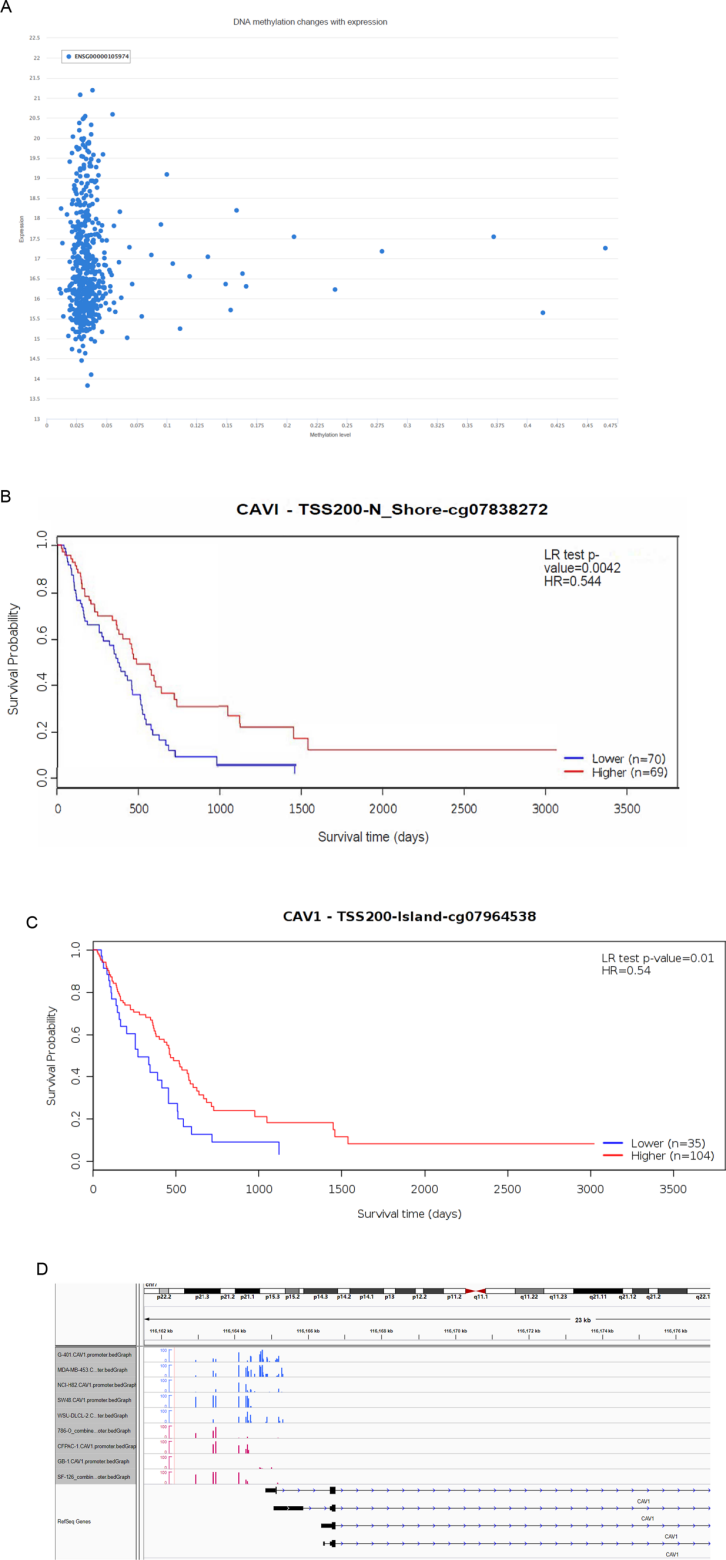
The methylation status of CAV1 and its relations with patients’ survival time. **A** The dot plot shows the expression of CAV1 and its methylation status. **B** The correlation between the methylation of CAV1 (at the location of Shore, cg-07838272) and patients’ survival, methylation probes cg-07838272. **C** The correlation between the methylation of CAV1 (at the location of TSS 200-Island, cg-07964538) and patients’ survival. **D** WGBS data of nine cell lines including two glioma cell lines (GB-1 and SF126) shows the methylation level in their DNA promoters

DNA methylation regulates gene expression and cancer progression [31]. Then we checked the methylation status of CAV1 in cancer and normal samples with the consideration of several factors including gender, pathological stages, copy numbers, etc. (Supplementary Fig. 3A). The cluster analysis of the methylation of CAV1 in four different transcripts was analyzed, As shown in Supplementary Fig. 3B (LGG) and Fig. 3C (GBM), the methylation level in GBM is higher than that in the LGG, there is no significant differences among the four different transcripts. We also checked the methylation status of CAV1 promoters and the correlation between the methylation level of CAV1 and the glioma patient’s survival time. As shown in Fig. 6A, the DNA methylation of CAV1 is very low and the expression of CAV1 is quite high. The same results were found in CGGA data too (Supplementary Fig. 4). The methylation level is negatively associated with the glioma pathological stages (Supplementary Fig. 4). Patients with lower methylation of CAV1 shows shorter survival (Fig. 6B, C and Supplementary Fig. 5).

According to our Whole Genome Bisulfite Sequencing (WGBS) of four cancer cells that are resistant to oxidative phosphorylation (786-O, CFPAC-1, GB-1, and SF126) and five types of cancer cells that are sensitive to OXPHOS inhibition (NCI-H82, G-401, MDA-MB-453, WSU-DLCL2, and SW48), the methylation of CAV1 in two glioma cancer cell lines (GB-1 and SF126) are very low compared to that in the resistant cell line group (Fig. 6D).

### High expression of CAV1 renders the glioma cells OXPHOS inhibition

**Fig. 7:**
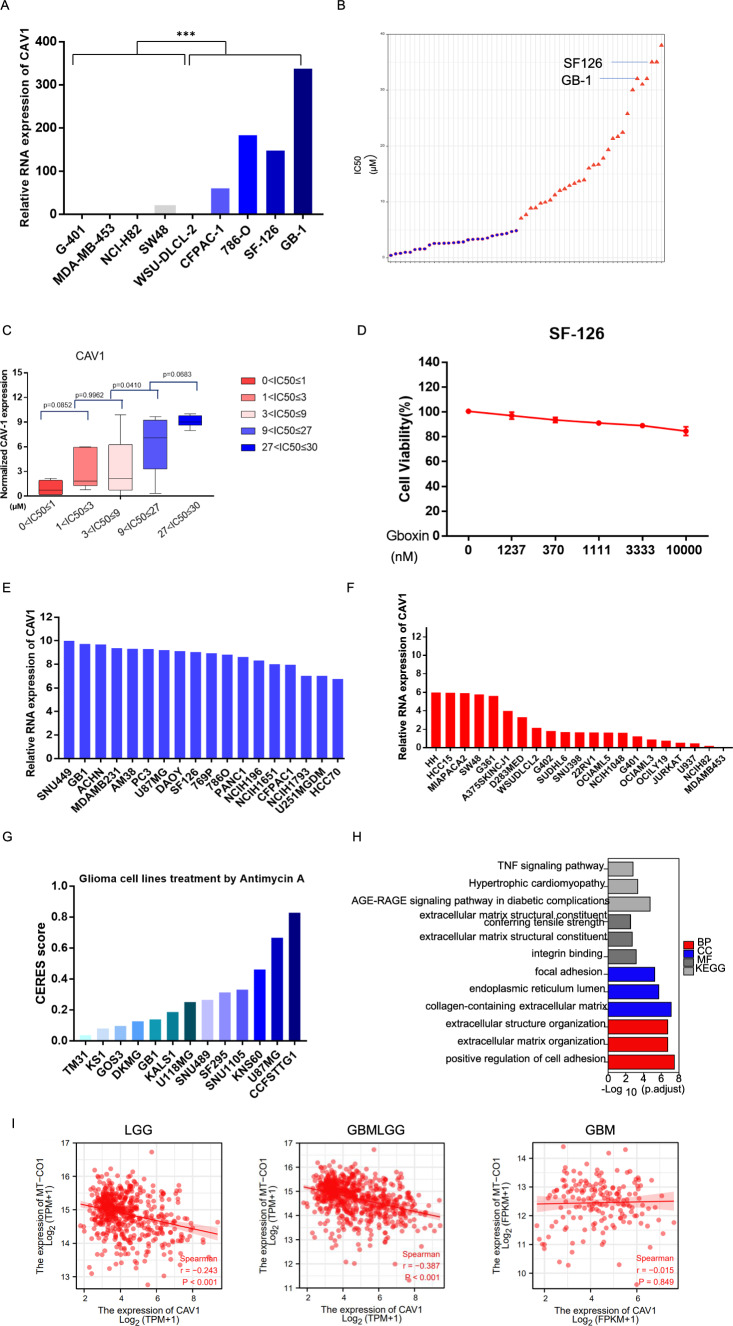

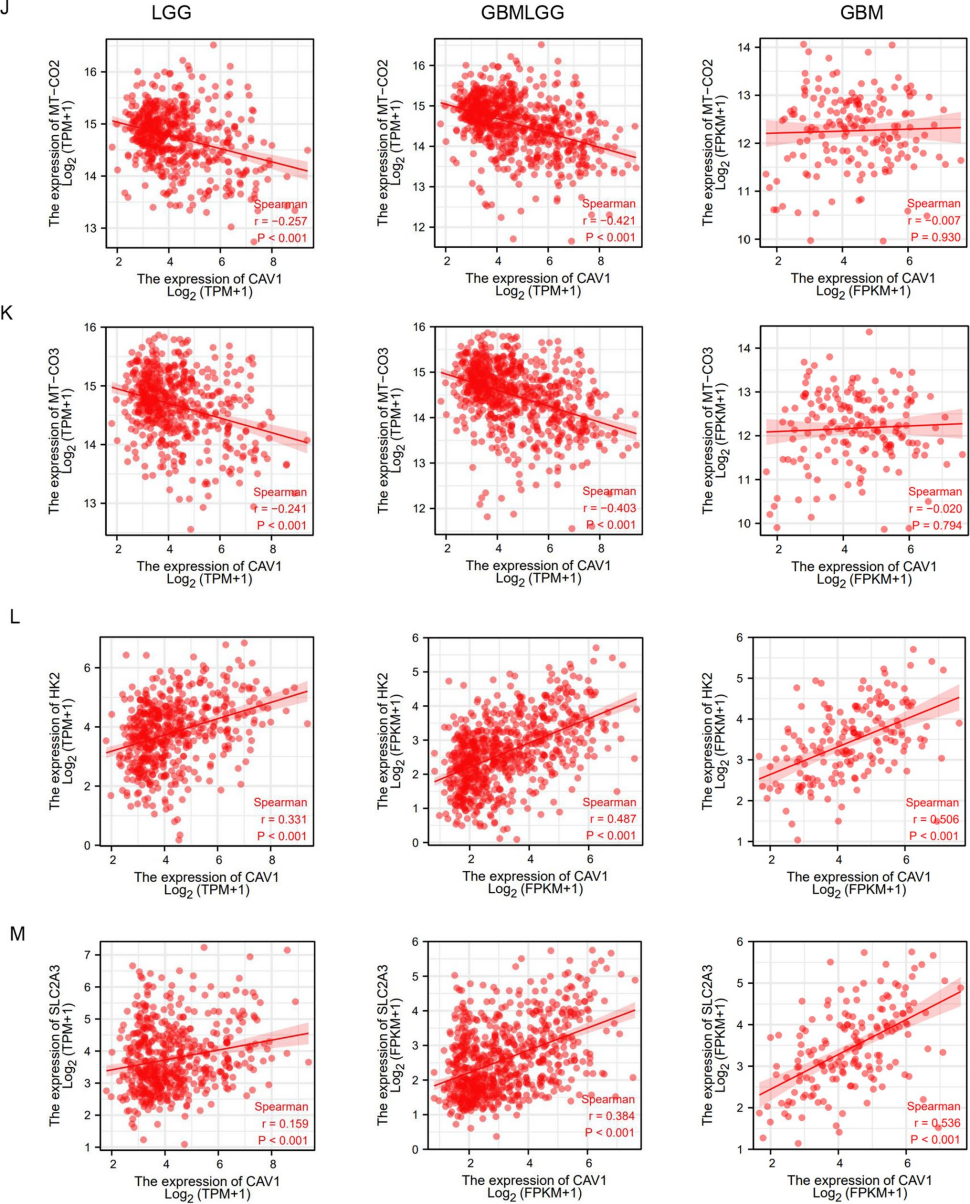
High expression of CAV1 renders the glioma cells OXPHOS inhibition. **A** Relative RNA expression of CAV1 in 9 cancer cells that we did RNA-sequencing, among which GB-1 and SF-126 are the human glioma cells. **B** The IC50 of all 57 cancer cell lines after treatment of Gboxin (the OXPHOS inhibitor) for 72 h. Glioma cancer cells SF-126 and GB-1 were marked. **C** The correlation between the expression of CAV1 and the OXPHOS-resistant cancer cell’s IC50. **D** Cell Viability test for SF-126 human glioma cancer cell line under Gboxin treatment in different dosages in 72 h. **E** The relative expression of CAV1 of OXPHOS-inhibition resistant cancer cell line, data from CCLE. **F** The relative expression of CAV1 of OXPHOS-inhibition sensitive cancer cell line, data from CCLE. **G** DepMap PRISM data shows more glioma cells under treatment with OXPHOS inhibitor Oligomycin A. The CERES scores were marked. **H** The GO/KEGG analysis of high-expressed genes in OXPHOS-inhibition groups. **I** The correlation between the expression of CAV1 in LGG gliomas with key OXPHOS genes including mt-CO1, mt-CO2 and mt-CO3. **J** The correlation between the expression of CAV1 in GBMLGG gliomas with key OXPHOS genes including mt-CO1, mt-CO2 and mt-CO3. **K** The correlation between the expression of CAV1 in GBM gliomas with key OXPHOS genes including mt-CO1, mt-CO2 and mt-CO3. **L** The correlation between the expression of CAV1 and HK2 in gliomas. **M** The correlation between expression of CAV1 and GLUT3 (SLC2A3) in gliomas. *p value < 0.05; **p value < 0.01;***p value < 0.001

Previous studies indicated that CAV1 is involved in the modulation of cancer metabolism and glycolytic activities [32]. In our RNA-sequencing data, we found CAV1 is much higher expressed in the OXPHOS-inhibition resistant groups than that in the sensitive groups (Fig. 7A). We used OXPHOS inhibitor Gboxin to treat all 57 cancer cells including glioma cancer cell GB-1 and SF-126 and found that both GB-1 and SF-126 are resistant to Gboxin, their IC50s are among the highest in these 57 cancer cells (Fig. 7B, Supplementary Table 3). Further analysis has shown that the expression of CAV1 is positively correlated with the IC50 of Gboxin for GB-1 and SF-126 (Fig. 7C). We verified the above result by Cell Viability Assay of SF-126, the cell viability doesn’t change significantly after Gboxin treatment in different dosage (Fig. 7D), indicating that SF-126 is resistant to Gboxin. We also explored CAV1 expression in OXPHOS-inhibition resistant and sensitive cells from CCLE and found that CAV1 is highly expressed in the OXPHOS-inhibition resistant groups than that in the sensitive groups (Fig. 7E, F). In the Depmap PRISM database, cell viability of more different glioma cells treated by another classical OXPHOS inhibitor Antimycin A is available, the CERES score of more glioma cells treated by Antimycin A is above zero (Fig. 7G, Supplementary Table 4), indicating that there are more glioma cancer types are resistant to the OXPHOS inhibition besides the cells lines GB-1 and SF-126 we checked.

To check whether CAV1 is the essential gene for all glioma cancer cell’s viability, we mined the DepMap data for the whole-genome CRISPR knocking out CAV1 in 67 glioma cancer cell lines and found the CERES scores of 45 cell lines are above zero and 22 cell lines are below zero (Supplementary Table 5), indicating that CAV1 are essential for some of the glioma cells not for all and there is high heterogeneity of gliomas.

When we conduct the GO/KEGG analysis for the highly expressed genes in the OXPHOS-resistant groups, we found the most significant pathways are the “collagen-containing extracellular matrix” in the biological process (BP) and “positive regulation of cell adhesion” in the molecular function (MF) (Fig. 7H). Considering promoting cell migration is one of the important functions of CAV1, and the RNA-sequencing data of resistant cells including two glioma cancer cell lines, we propose that the high expression of CAV1 may render the glioma cells OXPHOS inhibition and promotes cancer cell progression. To further verify the hypothesis, we checked the expression of CAV1 and the key OXPHOS pathway genes including MT-CO1, MT-CO2 and MT-CO3, and found that the expression of CAV1 is negatively related to the expression of MT-CO1, MT-CO2 and MT-CO3 in LGG and GBMLGG (Fig. 7I–K), but the correlations between CAV1 and OXPHOS genes in GBM is not as strong as that in LGG and GBMLGG. Meanwhile, we checked the expression of CAV1 with the key glycolysis pathway genes HK2 and GLUT3(SLC2A3) in gliomas and found that the expression of CAV1 is positively related to HK2 (Fig. 7L) and GLUT3 (Fig. 7M).

## Discussion

In this study, we deciphered the function of CAV1 in glioma progression and drug resistance. We have demonstrated that CAV1 expression has a positive correlation with glioma patients’ outcomes and dig out its working mechanisms: (1) The expression of CAV1 has a positive relation with the progression of glioma and a negative relation with glioma patients’ survival. (2) CAV1 expression has significant relations with immune infiltration and functions in glioma immunosuppression. (3) CAV1 contributes to glioma invasion and positively correlates with glioma stemness. (4) The methylation level of CAV1 has a positive correlation with patients’ survival. (5) CAV1 maintains the glioma cells’ resistance to OXPHOS inhibition. Our results show that CAV1 plays a critical role in glioma progression, drug resistance and glioma patients’ immunotherapy. We provide robust evidence for underlying mechanisms linking CAV1 expression, methylation, immune infiltration and drug resistance.

As an essential constituent of caveolar membrane proteins, CAV1 primarily functions in homeostasis, caveolae formation, caveolae trafficking and signal transduction, more studies demonstrated that it plays a complex role in disease by regulating signal pathways, interacting with immune cells, and mediating drug resistance [33]. Therefore, CAV1 is an important pathophysiological factor in various types of cancer [34]. CAV1 promotes tumor invasion and cancer cell migration in gastric cancer, lung cancer, renal and breast cancers [35]. Mechanistically, CAV1 accelerates gastric cancer progression via up-regulating the epithelial to mesenchymal transition under hypoxic conditions [36]. A recent study found that CAV1 activation drives mitochondrial fission and cytoskeleton remodeling to promote breast cancer migration [37]. Moreover, CAV1 also increases cancer progression by inhibiting ferroptosis in head and neck squamous cell carcinoma [38, 39]. CAV1 also interacts with other proteins to affect glioma cancer progression, it interacts with TRAF4, an E3 ubiquitin ligase, to maintain its deubiquitylation and stability, thus driving glioma stemness and Temozolomide resistance [40].

Cancer immunotherapy and immune checkpoint inhibition has become advanced therapy [41]. Understanding the characteristics of tumor-infiltrating immune cells in the glioma and the related co-working genes determines the cancer immunotherapy implications in cancer immunotherapy. CAV1 expression is significantly related to immune cell infiltration in glioma. Pharmacologically blocking CAV1 restores the function of the tumor-associated macrophage and facilitates more successful immunotherapeutic strategies directed against glioblastoma [42]. However, till now, there is no clinical trials in targeting CAV1 in glioma cancer immunotherapy.

Regarding to the role of CAV1 in drug resistance, CAV1 has been found to be upregulated in drug-resistant colon cancer [43], breast cancer [44] and lung cancers [45]. CAV1 is also a potential causative and critical factor in mediating trastuzumab resistance [46]. CAV1 interacts with P-glycoprotein encoded by the multidrug resistance-1 (MDR-1) gene located at the cell membrane [47]. In addition, CAV1 was highly expressed in cancer stem cells (CSCs) and modulated CSCs’ chemosensitivity. CAV1 deficiency sensitized breast CSCs by restricting their self-renewal ability by accelerating the differentiation process [33]. Our previous study found that cancer cells’ energy resource and reliance varies a lot [12], here we found that CAV1 is highly involved in the glycolysis and OXPHOS-inhibition drug resistance, which partially elucidate the working mechanisms of CAV1 in gliomas.

## Conclusion

In conclusion, we found that the scaffolding membrane protein CAV1 is highly expressed in glioma patients and predicts a poor prognosis, its promoters are hypomethylated and the methylation level is positively related with the glioma patients’ survival. CAV1 also contributes to glioma cancer stemness, cancer cell invasion, immune infiltration and cancer cells’ resistance to OXPHOS-inhibition drugs. Our research for the first time comprehensively studied the potential functions of CAV1 and provides insights on that CAV1 is a potential novel therapeutic target in gliomas.

### Supplementary Information

Below is the link to the electronic supplementary material.

Supplementary Table 1The DEGs in CAV-1-up and low-expressed groups in glioma patients from TCGA database. (XLSX 3129 KB)

Supplementary Table 2The GSEA analysis of the DEGs in CAV-1 high and low groups in glioma patients from TCGA database (XLSX 43 KB)

Supplementary Table 3The IC50s of 57 cancer cell lines treated by Gboxin. (XLSX 14 KB)

Supplementary Table 4Glioma cells treated with Oligomycin A, data from DepMap. (XLSX 10 KB)

Supplementary Table 5Cell viability of CRISPR knocking out CAV1 in 67 glioma cell lines, data from DepMap. (XLSX 66 KB)

Supplementary Figure 1 The expression of CAV1 in Chinse glioma patients (CGGA database).(A). The expression of CAV1 in different histology of Chinese gliomas. (B). The expression of CAV1 in different pathological stages in Chinese glioma patients. (C) The expression of CAV1 in IDH-mutant and IDH-wild type gliomas in Chinese patients. (D). The expression of CAV1 in different grades in IDH-mutant and IDH-wild type gliomas in Chinese patients. (E). The expression of CAV1 in different genders of Chinese glioma patients. (F). The expression of CAV1 in different ages of Chinese glioma patients. (G). The expression of CAV1 in primary and recurrent gliomas of Chinese patients. (H). The expression of CAV1 in different stages of primary and recurrent gliomas of Chinese patients. Supplementary Figure 2 Correlation between the expression of CAV1 and the Chinese glioma patients’ survival (CGGA database).(A). Correlation between the expression of CAV1 and the Chinese glioma patients with all different grades of gliomas. (B). The correlation between the expression of CAV1 and the survival of primary glioma and recurrent gliomas in grade II in Chinese patients. (C). The correlation between the expression of CAV1 and the survival of primary glioma and recurrent gliomas in grade III in Chinese patients. (D). The correlation between the expression of CAV1 and the survival of primary glioma and recurrent gliomas in grade IV in Chinese patients.Supplementary Figure 3 The methylation level of CAV1 in glioma and normal samples (TCGA database).(A). The methylation of CAV1 promoters in gliomas and normal samples, glioma patients in different stages. (B). Heatmap shows the methylation of 4 different transcripts in LGG samples (green color represents normal profiles, black represents disease profiles). (C). The result heatmap contains methylation data of 4 transcripts of CAV1 from 155 samples of 450 k. In the heatmap, rows represent transcripts and columns represent samples (green color represents normal profiles, and black represents disease profiles). Supplementary Figure 4 The methylation of CAV1 in CAV1 (CGGA database).(A). The methylation of CAV1 of glioma patients with different histology. (B).The methylation level of CAV1 in different pathological stages. (C). The methylation of CAV1 in different genders in different stages. (D). The methylation level of CAV1 in glioma patients of different ages.Supplementary Figure 5 The correlation between the methylation level and the patients’ survival (CGGA database)(A). The correlation between the methylation level of CAV1 and patients’ survival. (B). The correlation between the methylation level of CAV1 and patients survival for patients in grade II (left) and grade III (right) stages. (C). The correlation between the methylation level of CAV1 and survival for patients in grade IV. (PDF 2218 KB)

## Data Availability

The data underlying this study are freely available from TCGA data portal (https://portal.gdc.cancer.gov/projects/TCGA-LGG) and GEO dataset (http://www.ncbi.nlm.nih.gov/geo/). The RNA-seq and WGBS raw sequence data reported in this paper have been deposited into the Genome Sequence Archive (GSA) for humans under accession: HRA001452.
