# Supplementary material for: Caveolin-1 promotes glioma progression and maintains its mitochondrial inhibition resistance
Source: Discov Oncol. 2023 Aug 29;14:161. doi: 10.1007/s12672-023-00765-5 (PMC10465474; doi:10.1007/s12672-023-00765-5)

Supplementary Figure 1

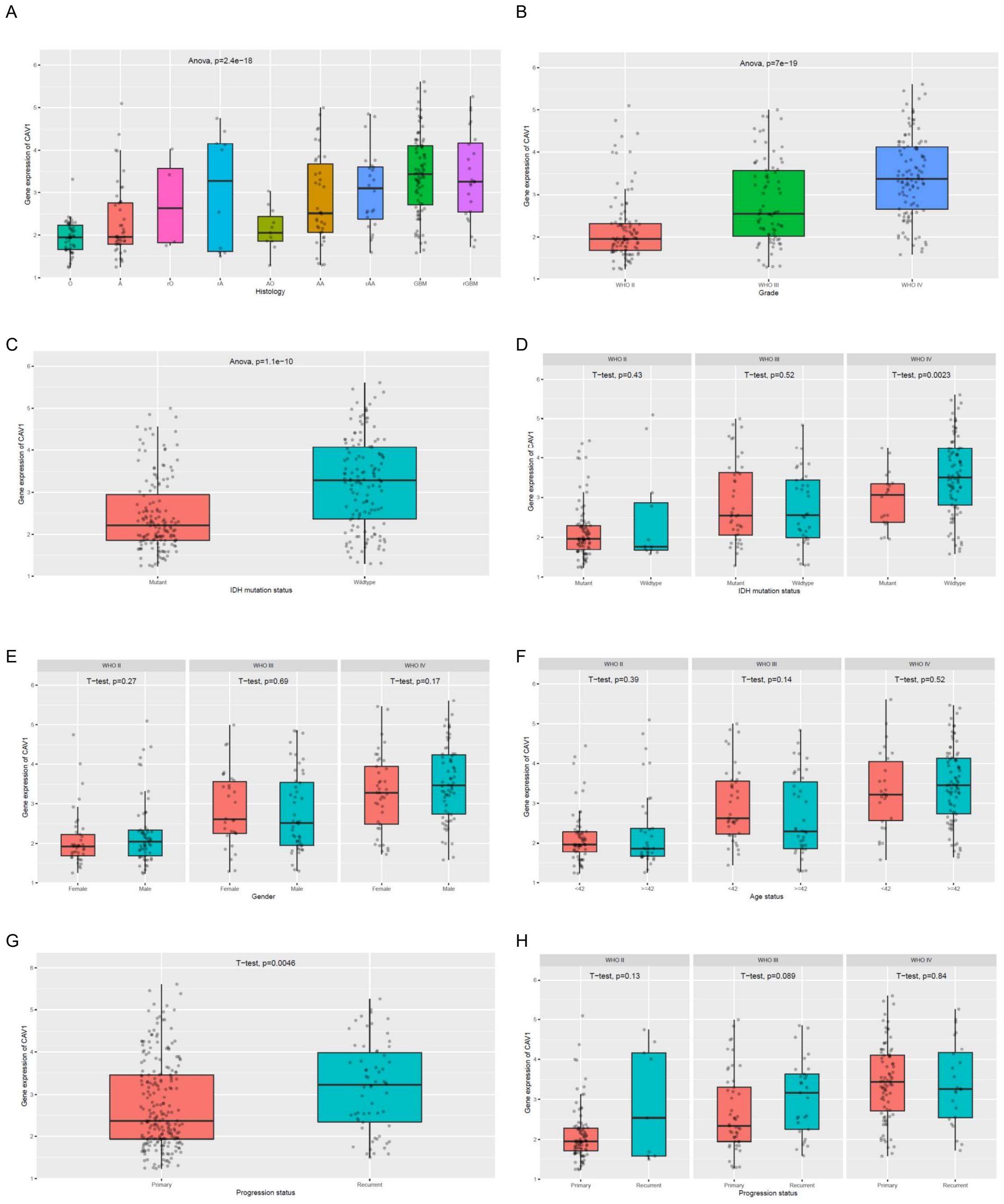

Supplementary Figure 2

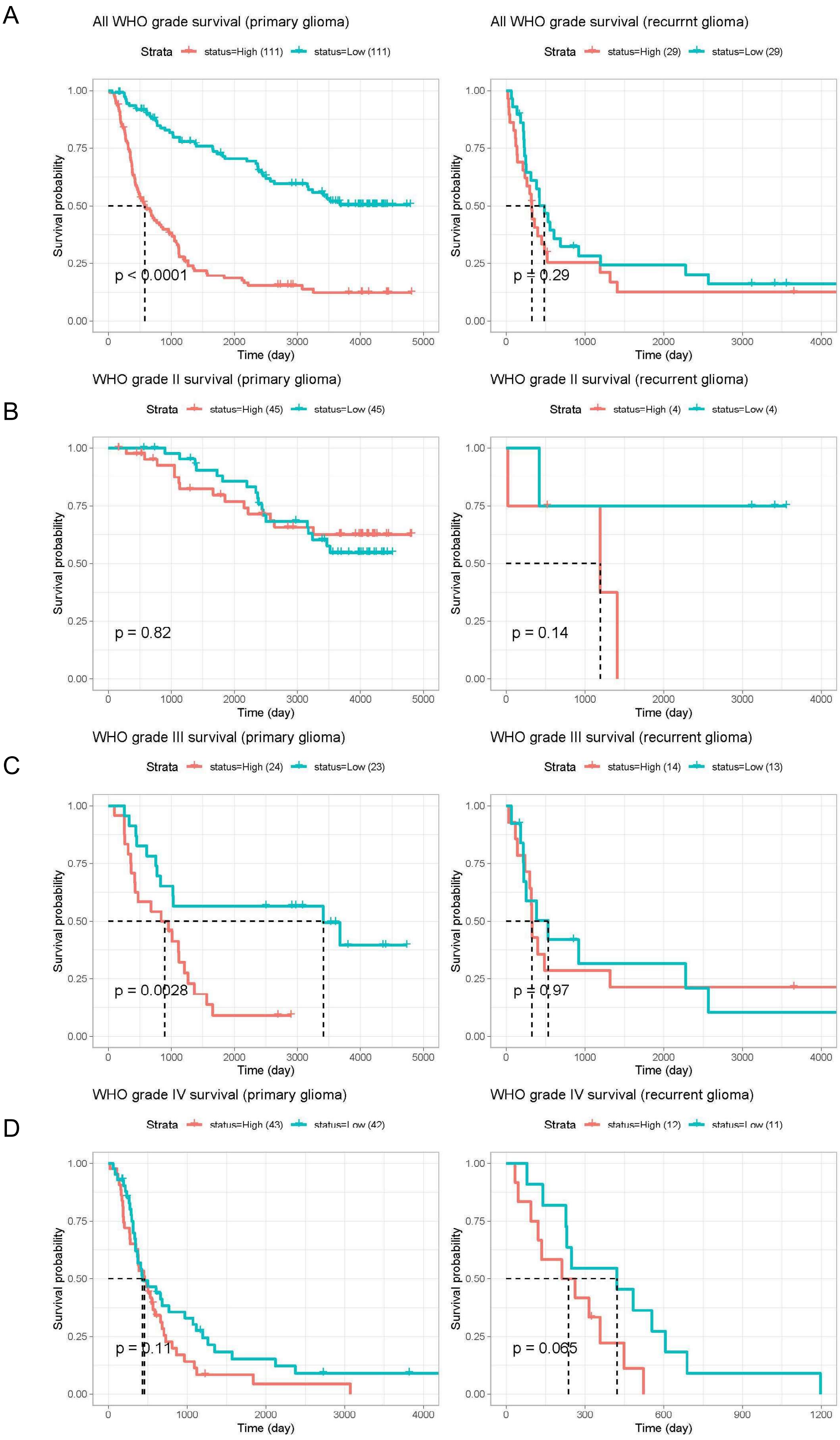

Supplementary Figure 3

A

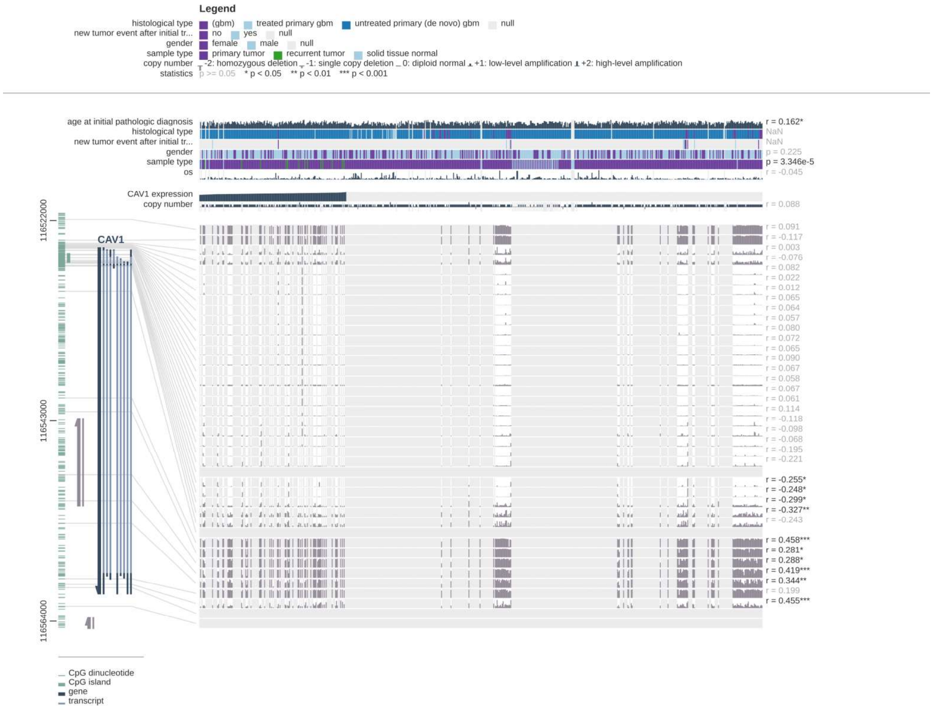


B

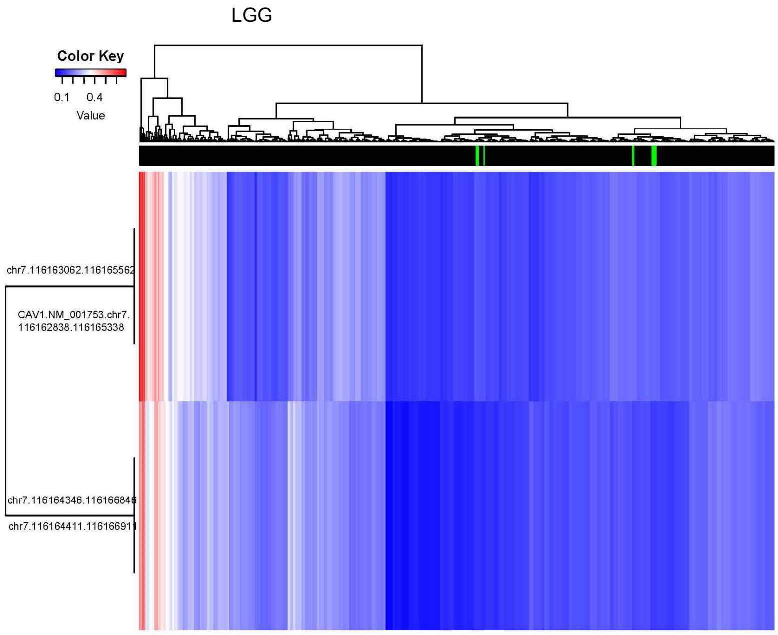


C

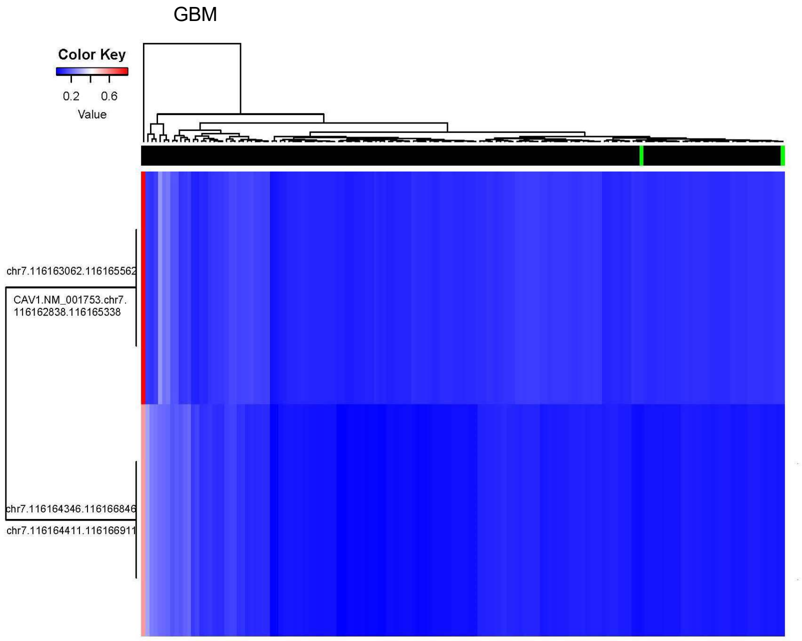

Supplementary Figure 4

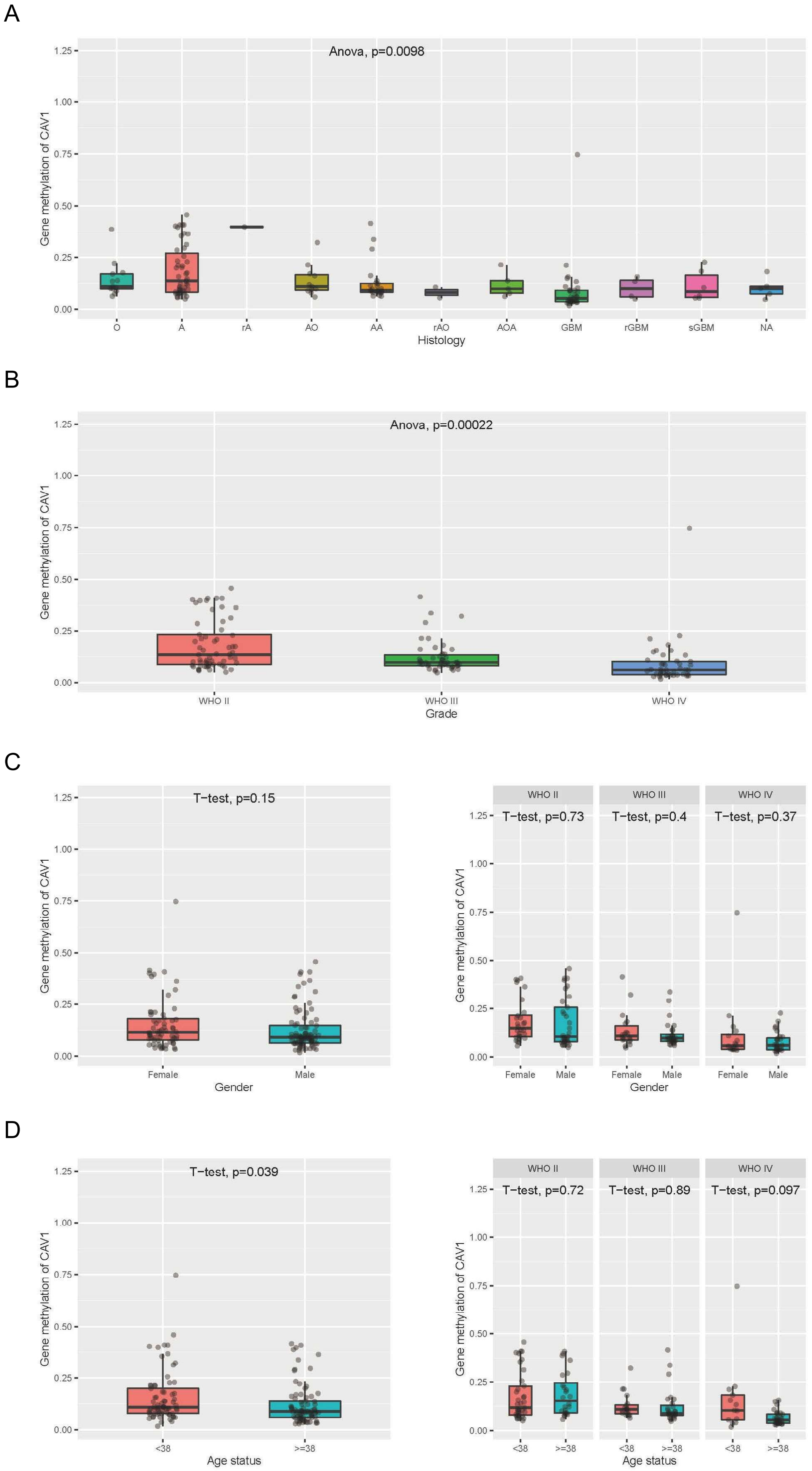

Supplementary Figure 5

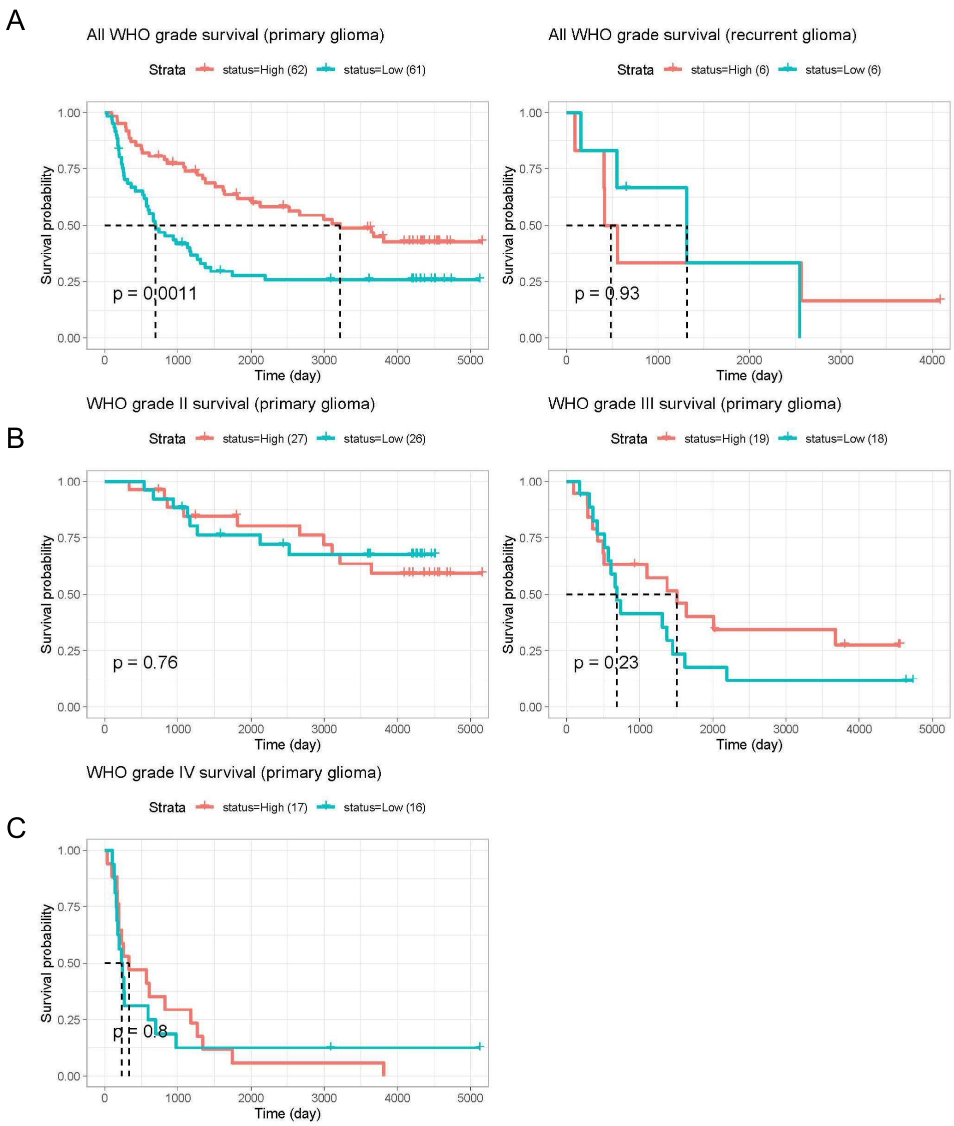

Supplement: Supplementary file 6 — Supplementary Figure 1 The expression of CAV1 in Chinse glioma patients (CGGA database).(A). The expression of CAV1 in different histology of Chinese gliomas. (B). The expression of CAV1 in different pathological stages in Chinese glioma patients. (C) The expression of CAV1 in IDH-mutant and IDH-wild type gliomas in Chinese patients. (D). The expression of CAV1 in different grades in IDH-mutant and IDH-wild type gliomas in Chinese patients. (E). The expression of CAV1 in different genders of Chinese glioma patients. (F). The expression of CAV1 in different ages of Chinese glioma patients. (G). The expression of CAV1 in primary and recurrent gliomas of Chinese patients. (H). The expression of CAV1 in different stages of primary and recurrent gliomas of Chinese patients. Supplementary Figure 2 Correlation between the expression of CAV1 and the Chinese glioma patients’ survival (CGGA database).(A). Correlation between the expression of CAV1 and the Chinese glioma patients with all different grades of gliomas. (B). The correlation between the expression of CAV1 and the survival of primary glioma and recurrent gliomas in grade II in Chinese patients. (C). The correlation between the expression of CAV1 and the survival of primary glioma and recurrent gliomas in grade III in Chinese patients. (D). The correlation between the expression of CAV1 and the survival of primary glioma and recurrent gliomas in grade IV in Chinese patients.Supplementary Figure 3 The methylation level of CAV1 in glioma and normal samples (TCGA database).(A). The methylation of CAV1 promoters in gliomas and normal samples, glioma patients in different stages. (B). Heatmap shows the methylation of 4 different transcripts in LGG samples (green color represents normal profiles, black represents disease profiles). (C). The result heatmap contains methylation data of 4 transcripts of CAV1 from 155 samples of 450 k. In the heatmap, rows represent transcripts and columns represent samples (green color repre [file 12672_2023_765_MOESM6_ESM.pdf]
